# Increase Intensive Care Staff Comfort and Proficiency With Emergent Re-sternotomy in the Post-Open-Heart Patient by Using SynDaver® Simulation

**DOI:** 10.7759/cureus.20875

**Published:** 2022-01-02

**Authors:** Darlene R Deters, John Hunninghake, Judy Ruiz, Deborah J Marquez, Deborah J Ramirez, Robert V Coffman

**Affiliations:** 1 Center for Nursing Science and Clinical Inquiry, Brooke Army Medical Center, Fort Sam Houston, USA; 2 Pulmonary and Critical Care Medicine, Brooke Army Medical Center, Fort Sam Houston, USA; 3 Cardiovascular Intensive Care Unit, Brooke Army Medical Center, Fort Sam Houston, USA; 4 Education, Brooke Army Medical Center, Fort Sam Houston, USA; 5 Simulation, Brooke Army Medical Center, Fort Sam Houston, USA

**Keywords:** proficiency, emergent re-sternotomy, syndaver®, simulation training, comfort

## Abstract

Simulation training has been used in many avenues such as aeronautics, law enforcement, and healthcare to assist in training personnel to learn a new task and perform highly technical procedures. Simulation training has demonstrated beneficial for providing low-use, high-risk jobs such as landing a plane with a complete engine failure, performing reconstructive surgery, and even emergent lifesaving procedures. Our simulation training group chose to develop our custom hands-on training to perform emergent re-sternotomy on the post-open-heart patient based upon this belief. The goal of this project was to assist the bedside intensive care nurse in their self-perception of being comfortable and proficient in helping the physician with the procedure of an emergent re-sternotomy on the post-surgical open-heart patient. Measurement of self-perception of comfort and proficient was measured with a pre/post-questionnaire. The pre/post-questionnaire results showed improvement ranging from an increase in self-scoring from 1.2 to 1.7, with statistical significance demonstrated with a p <0.05.

## Introduction

Simulation-based training has become a basic standard in the medical field for training medical staff in procedures from the basic to the more advanced medical procedures. Simulation is also used to train medical staff in low-volume, high-risk techniques in the hopes that they will never have to perform an emergent re-sternotomy [1,2]. Still, if they must, they will at least have some level of familiarity with the procedure. As Benner describes, the Dreyfus model of skill acquisition states that there are five levels of proficiency that one must pass through to achieve independent proficiency [3,4]. The five levels are novice, advanced beginner, competent, proficient, and expert. The staff who care for the cardiothoracic (CT) surgical patients are predominantly at the levels of proficient and expert.

The question that has arisen regarding our institution is the following; nurses classified as an expert have to perform or assist in high-risk procedures that they have never had to participate in beforehand. Secondly, with the constant turnover of our advanced beginner or competent nurses, the best method for the staff to achieve comfort in performing low-volume high-risk procedures. The use of simulation-based skill training has gained in popularity and as a standard of care for developing formative and a summative assessments of the necessary skills needed for a positive learning experience in cardiac surgery residency programs [5,6]. Furthermore, as in many circumstances with simulation training, the closer the simulation is to the real world, the more positive the learning experience is for the trainees as they maintain critical skills.

The intent of developing this novel training program is to focus on the importance of the role of the nursing personnel and the task of assisting with performing an emergent re-sternotomy. Acquiring new knowledge with this training program will provide additional skills to assist surgeons with this emergent procedure. This training program also intends to assist the staff in acquiring confidence in their ability to function independently. Also, to operate safely, this procedure may need to be performed with a cardiothoracic (CT) surgeon or with a less experienced trauma surgeon when the CT surgeon is on their way to the unit.

For simulation-based training to be effective, the training needs to be as natural to real life as possible [4-6]. Unfortunately, our institution’s simulation equipment did not meet that need, as true to life as possible, for several reasons. The first issue was about electrical safety for our staff. Secondly, internal organs needed to be simulated in some fashion that would exhibit a sense of realism. Lastly, financially, the simulation device needed to be used repeatedly without losing the actual effect (or process) of performing an emergent sternotomy [7-10]. After exploring the market, we decided on a SynDaver® custom model, which included the ability to repeatedly simulate a re-sternotomy into the chest cavity without jeopardizing the integrity of the anatomic model.

Cardiac tamponade or hypovolemia (bleeding) after cardiac surgery can delay appropriate cardiopulmonary resuscitation (CPR) due to a lack of perfusion. In situations where external cardiac compression will be of no effect and emergent re-entry into the chest cavity needs to occur. The Society of Thoracic Surgeons (STS) goal is to perform an emergent re-entry sternotomy within five minutes [11].

The STS has identified five key factors that should be established into a clinical protocol for institutions that care for post-cardiac surgery patients. The first of the key factors is that successful treatment of a patient who arrests after cardiac surgery should be rehearsed frequently, utilizing a team approach. The second key factor is that the team should be able to perform post-surgery ventricular fibrillation training by being able to perform three sequential defibrillations before external cardiac chest compressions are performed; if this fails, then be able to perform an emergent re-sternotomy. The third key factor for the institution to perform is immediate treatment of post-surgery asystole or extreme bradycardia by attempting pacing before external cardiac chest compressions are performed; if pacing fails, perform an emergent re-sternotomy. The fourth key factor to be addressed is if pulseless electrical activity occurs, and that cannot be quickly reversed, warrants an emergent re-sternotomy. The final key factor that should be understood and be part of training is that full-dose use of epinephrine should be avoided due to the probability of extreme hypertension, resulting in loss of the graft.

## Materials and methods

Plan implementation

After much discussion with our institution’s simulation department, the physical properties of the SynDaver® device appeared to meet our needs best. Therefore, in consultation with the SynDaver® engineers, our physician group, the simulation department, and nursing personnel, a plan was developed, and the financial support process was initiated.

**Figure 1:**
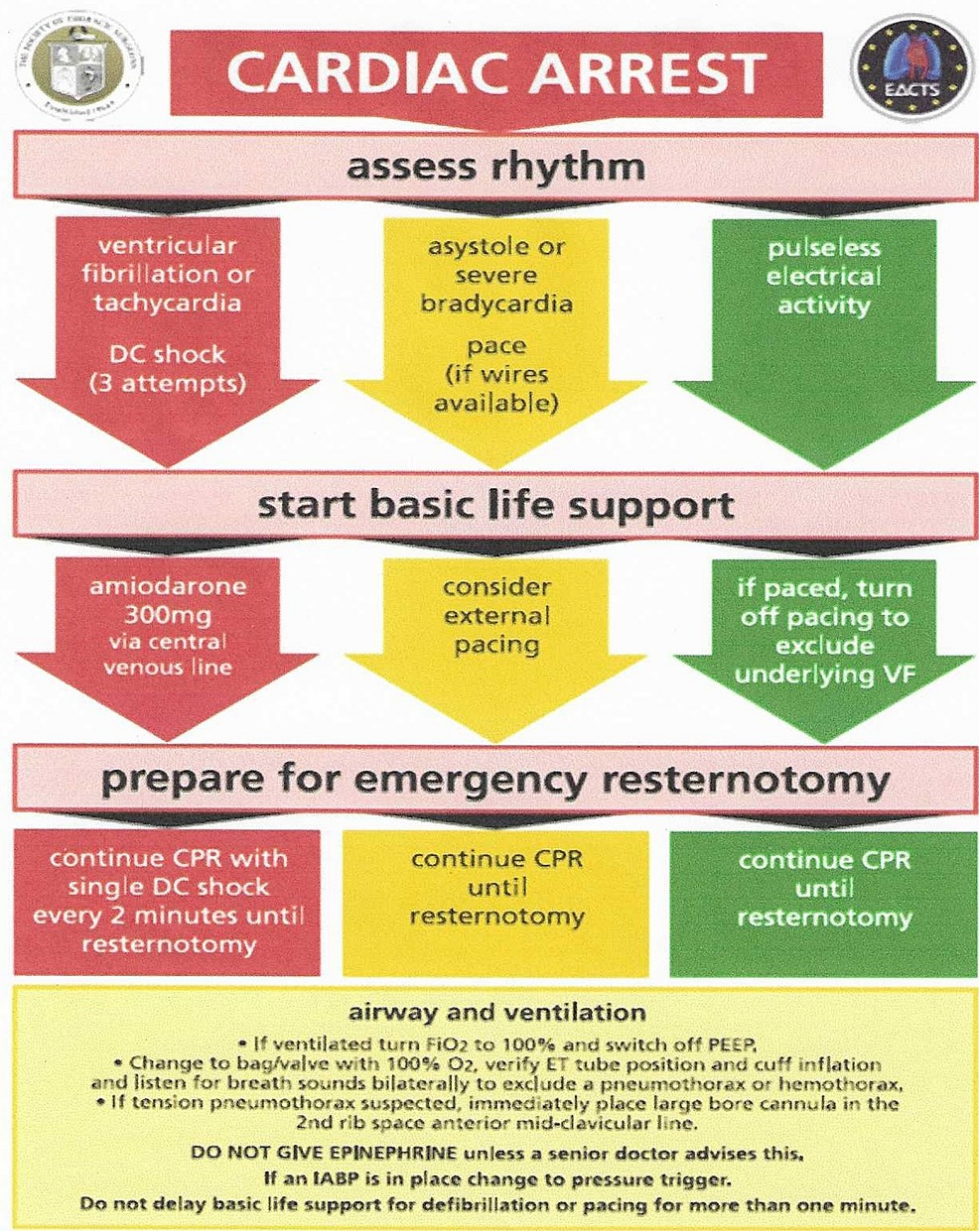
Society of Thoracic Surgeons algorithm. Used with permission [11]. DC: direct current, CPR: cardiopulmonary resuscitation, VF: ventricular fibrillation, PEEP: positive end expiratory pressure, ET: endotracheal tube, IABP: intra-aortic balloon pump.

**Figure 2:**
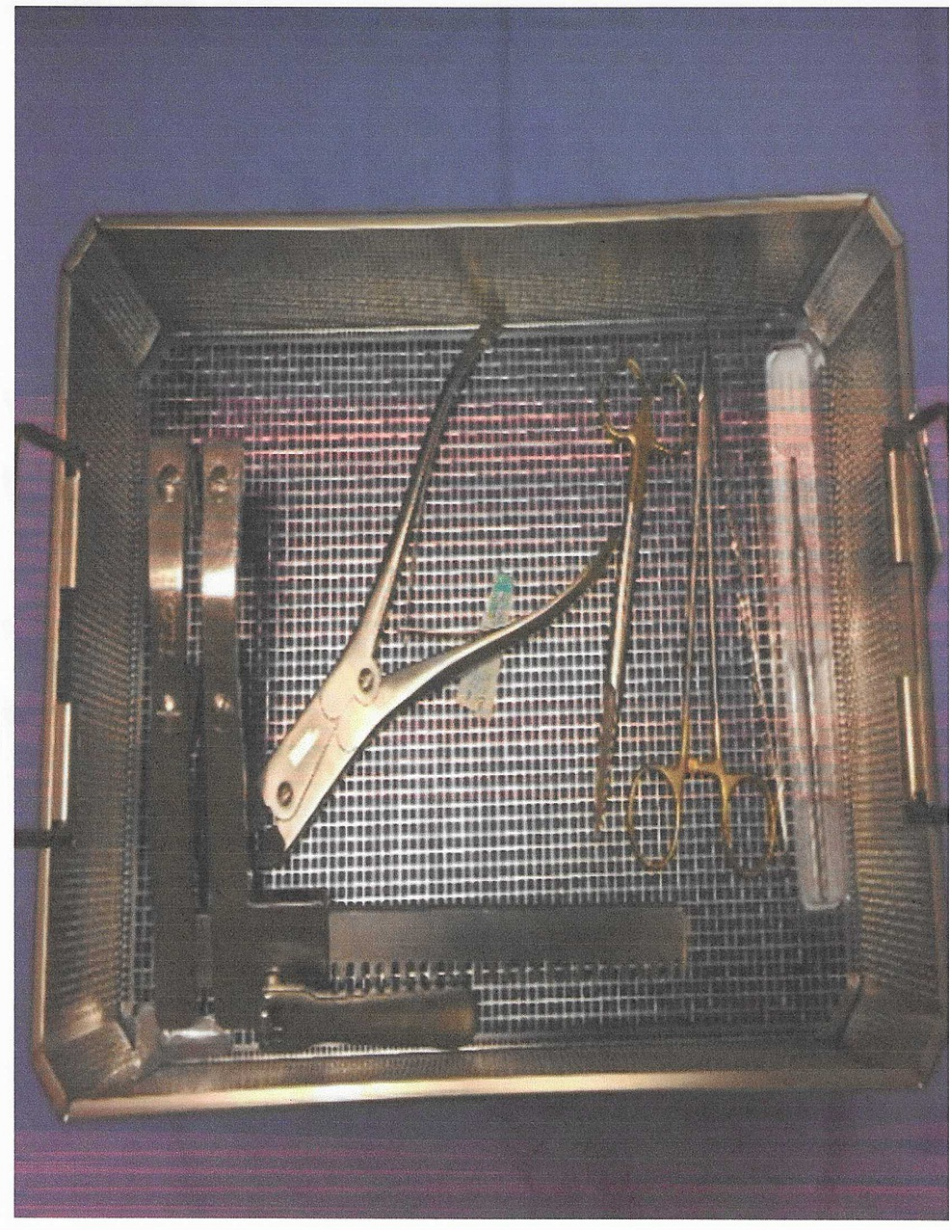
Emergent sternotomy surgical set-scaled down from original chest set.

**Figure 3:**
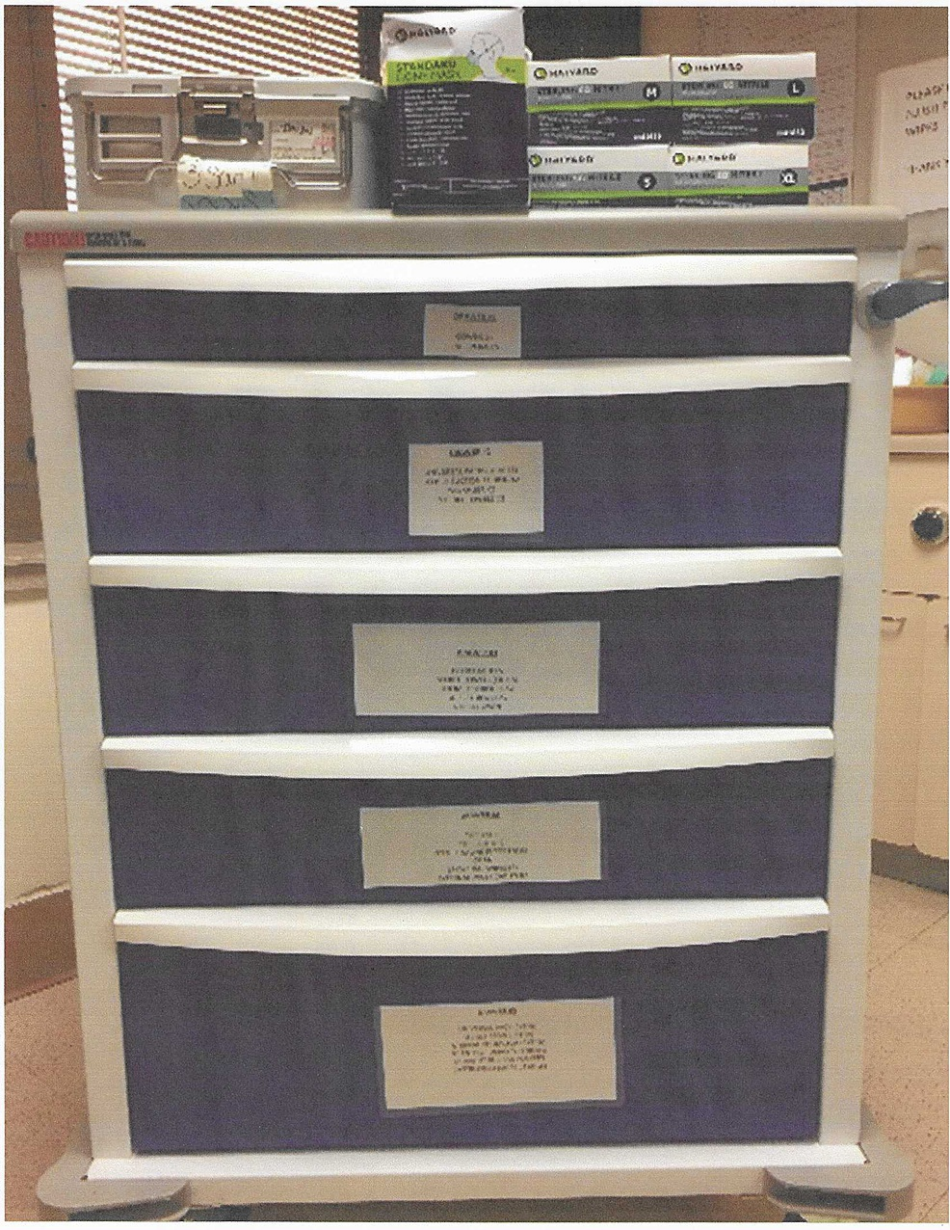
Dedicated emergent re-sternotomy cart with clearly label drawer contents.

The initial layout of the cart used for emergent sternotomy was quite extensive and was not intuitive to use. Since our simulation scenarios were modeled after the STS (Figure 1), we began organizing the sternotomy on their recommendations. With input from the cardiovascular surgeon group and the operating room nurse, the final decision was made to streamline the emergent re-sternotomy cart (Figures 2, 3). The new re-sternotomy cart was setup to be utilized in a top-down approach. The top of the cart contains first-line instruments and sterile gloves needed for the re-sternotomy procedure. After some episodes of simulation training, the cart contents progressed to extra items in the fifth drawer, such as additional sternal retractors (Table 1).

**Table 1:** Emergent sternotomy cart contents by drawer. CHG: chlorhexidine, CT: cardiothoracic.

Cart location	Content
Top of cart	Skin prep-large CHG scrubs, gloves-sizes 6½, 7, 7 ½, 8, 8½, small instrument tray consisting of #10 and #15 disposable blade, Mayo scissors, heavy needle driver, wirecutter, sternal retractor, sterile #22 blade, and blade handle
Side of cart	CT cart checklist, Zoll CT checklist, and caps and masks with face shield
Drawer #1	Gowns ×4 and lap sponges ×2
Drawer #2	Universal pack (drapes), sterile suction tubing ×2, Yankauer ×2, and sterile towels ×2
Drawer #3	Sterile #22 blade and blade handle, internal defib paddles, sterile towels (extra), Toomey syringes ×2, bulb syringes ×2, and sterile basin
Drawer #4	Sterile 5-in-1, sterile saline (bottles) ×2, Ioban, epicardial wires (4), and external pacer cables ×2
Drawer #5	Universal pack (extra), sterile basin (extra), sternal retractor (extra), 9" and 12" Debakey forceps, 6" and 8" Russian forceps, Castroviejo (small, large), and sutures (#3, 4, 5 prolene)

A pre-briefing was conducted regarding the emergent re-entry sternotomy class, and an open discussion was encouraged by all participants. The discussion was prefaced with the instruction that questions are welcome and important. We explained that this training is meant to create a non-judgmental environment to promote learning. In addition, this training was intended to cover the different roles and responsibilities of the participants in the actual process of emergent re-sternotomy. Our emergent re-entry protocol includes the following nursing roles.

**Figure 4:**
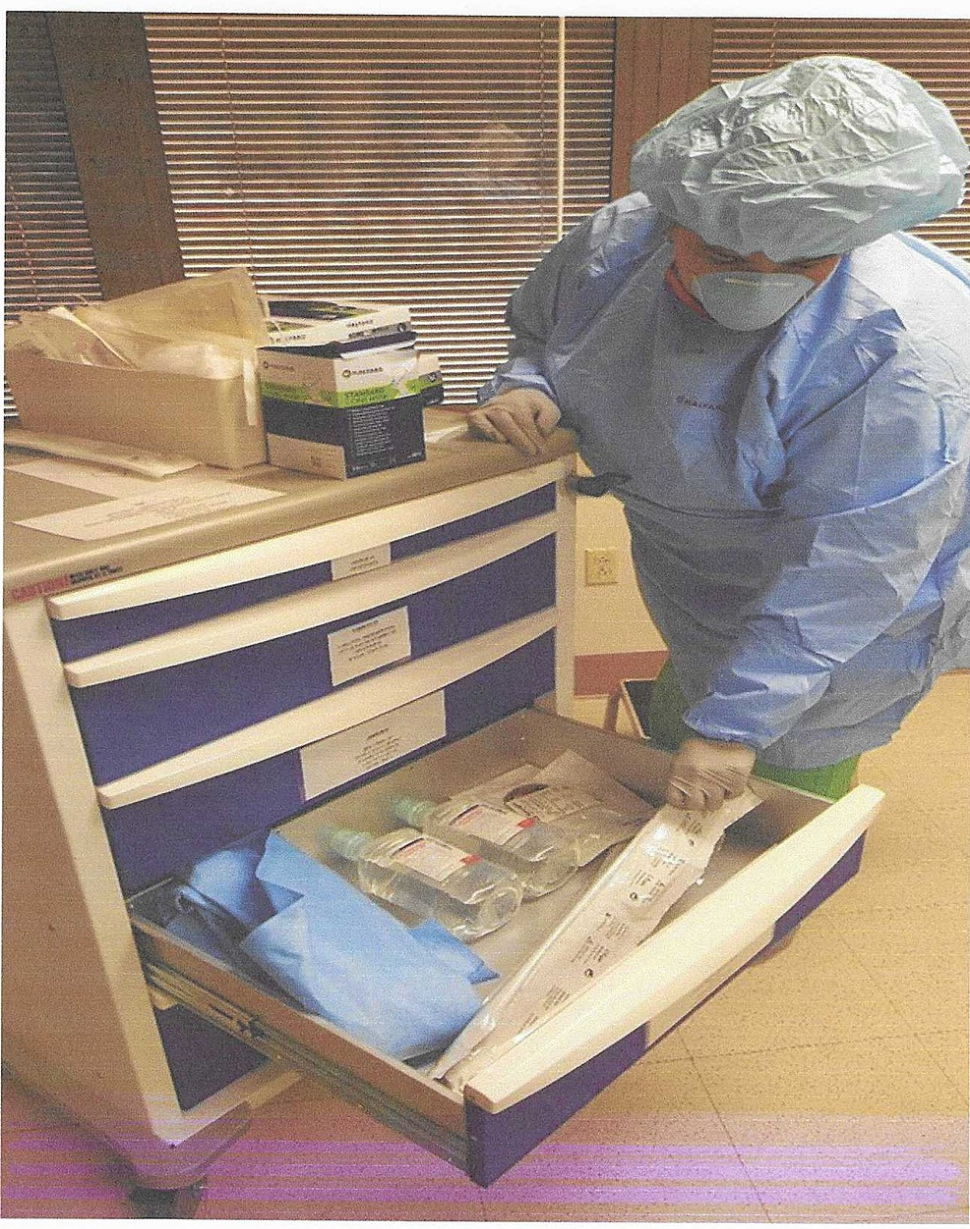
Dedicated role as a chest re-entry nurse.

Chest cart nurse-passes trays, drapes, etc., from cart to scrub nurse (Figure 4).

**Figure 5:**
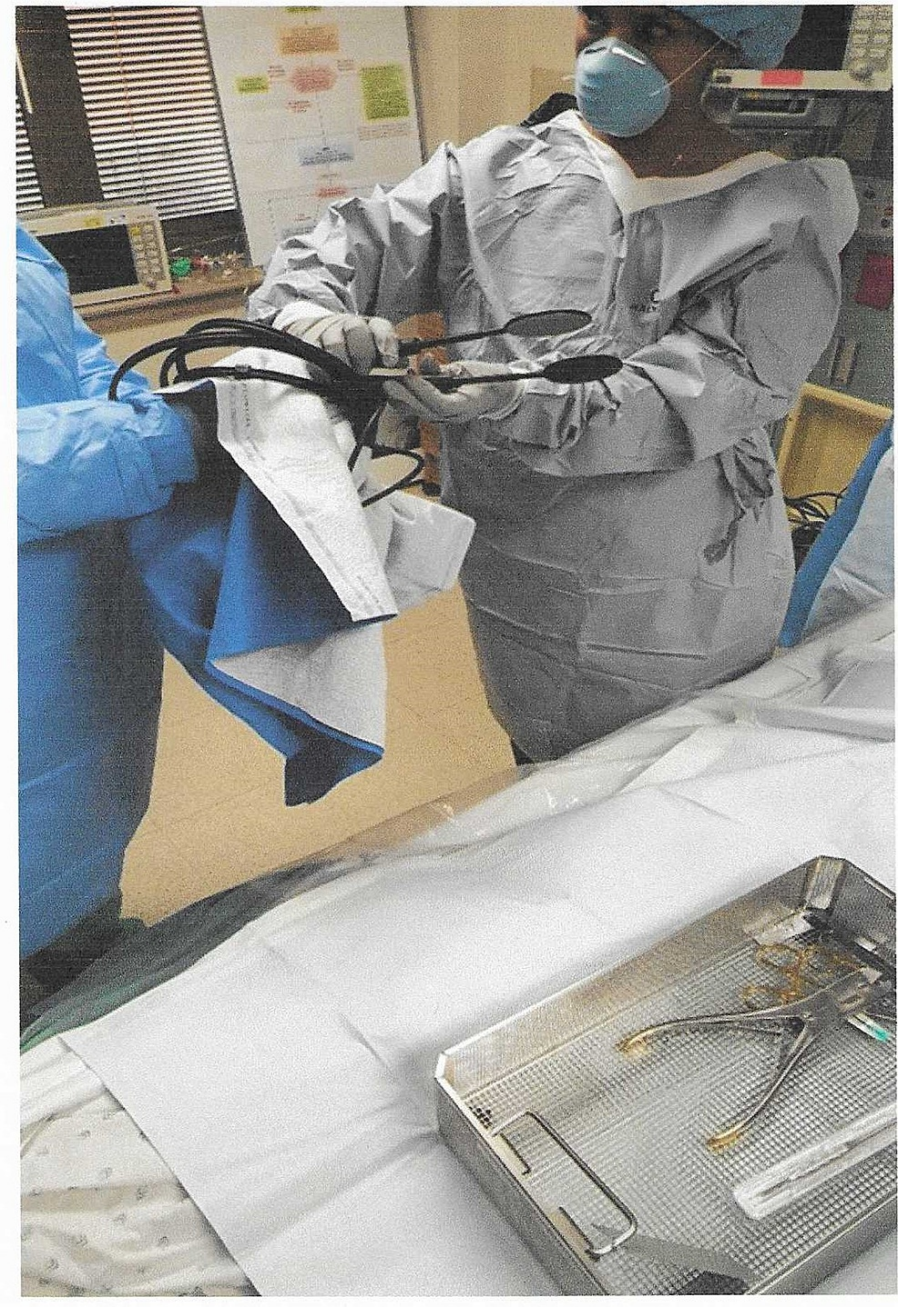
Circulating nurse handing over sterile equipment to the surgeon.

Circulating nurse-focuses on the patient, removing clothing, ECG pads from the operating area, connects suction, obtains and positions lights, checks and hangs blood/blood products (Figure 5).

Scrub nurse-(not the primary nurse)-remains sterile, establishes sterile field, assists the surgeon with gowning and gloving, draping the patient, opens the instrument trays, and passes the instruments to the surgeon.

Medication nurse-(must be an RN)-administers intravenous push (IVP) medications, helps to check blood/blood products, switches to internal paddles once the chest is entered.

Runner nurse-all-around assistant; goes to blood bank; fills in as needed.

**Figure 6:**
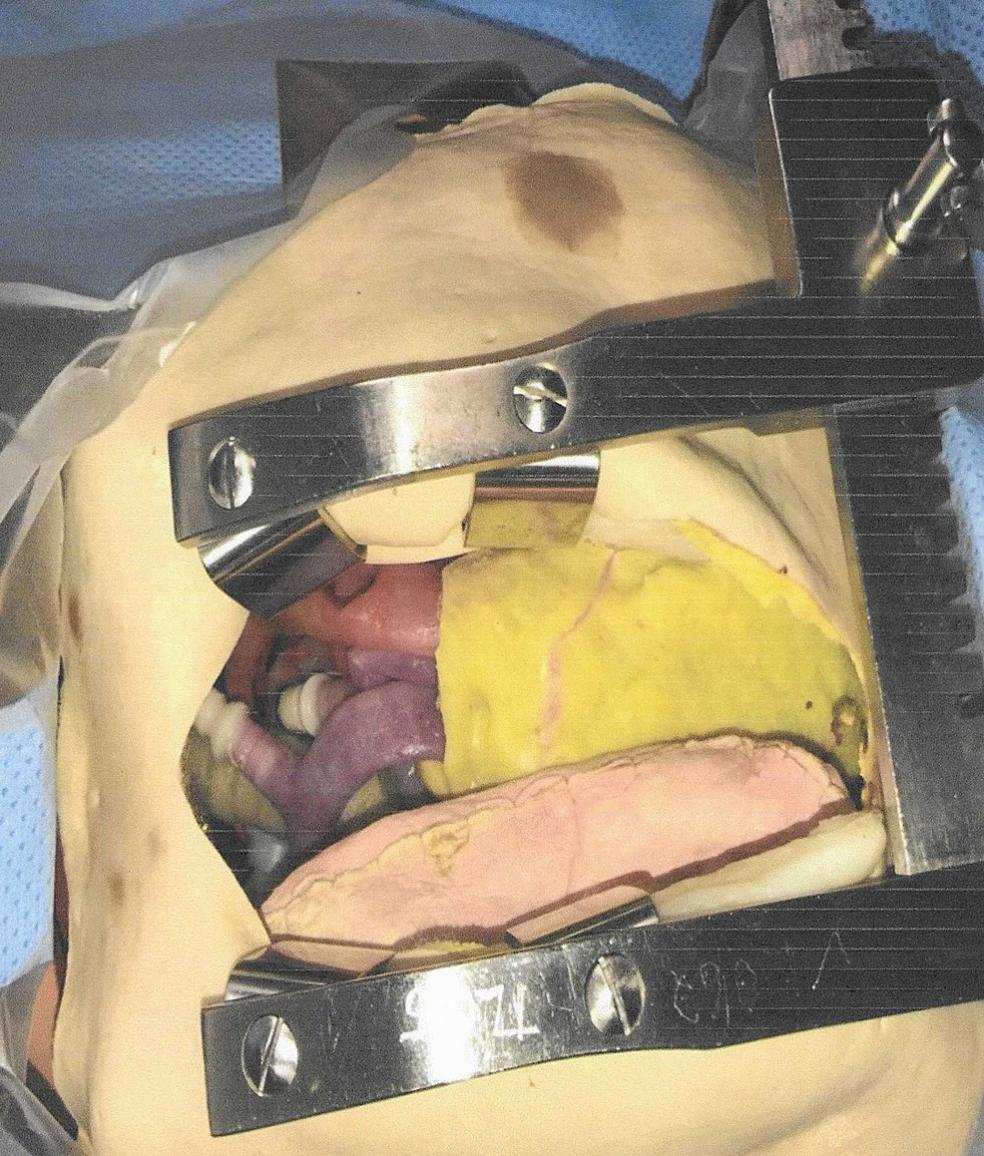
Simulated emergent sternotomy with chest spreader engaged.

**Figure 7:**
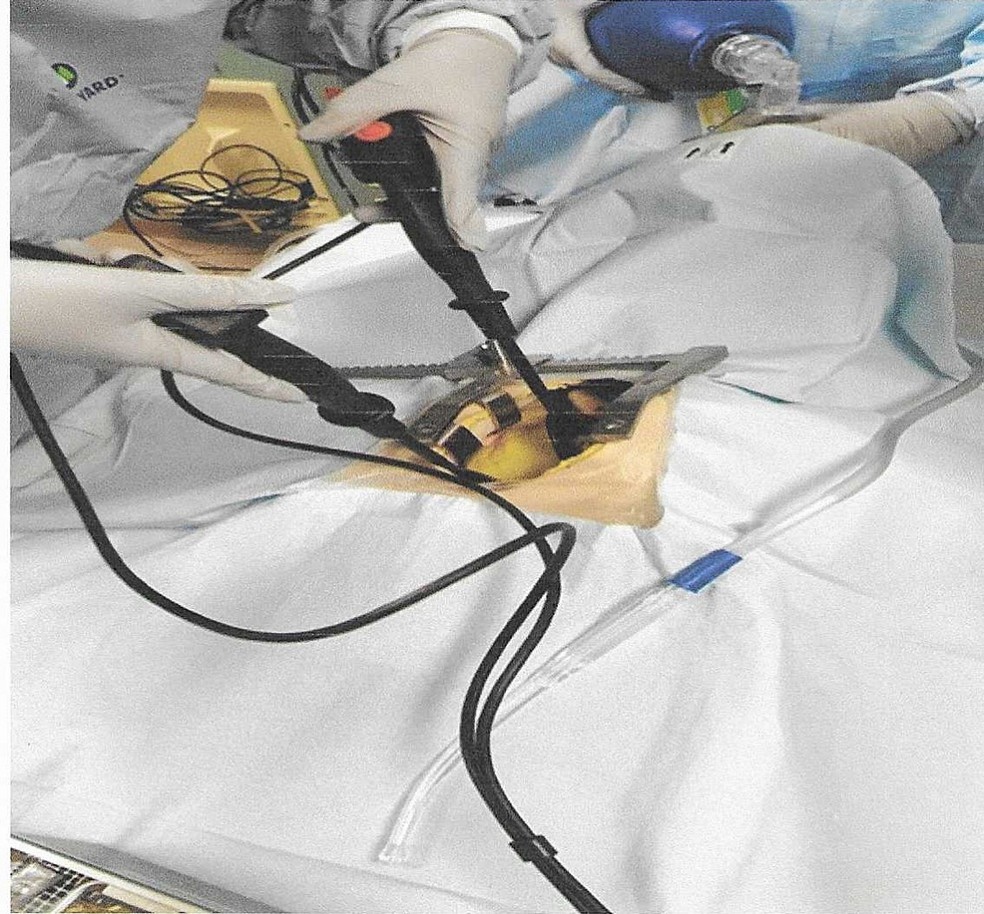
Simulating internal defibrillation.

Scenario-based training was completed with a multidisciplinary approach, including cardiothoracic surgeons, physicians, open heart nurses, and support nursing staff (Figures 6, 7). The two core scenarios utilized were for cardiac tamponade and R on T dysthymia. After the completion of the group scenario, debriefing and collegial discussion occurred. The debriefing focused on the reflection of the group’s overall performance and their performance within the scenario’s critical action. Any gaps identified by the participants generated an open discussion with possible solutions. The scenarios were repeated if the group felt this was warranted, but it was not mandatory. 

## Results

**Figure 8:**
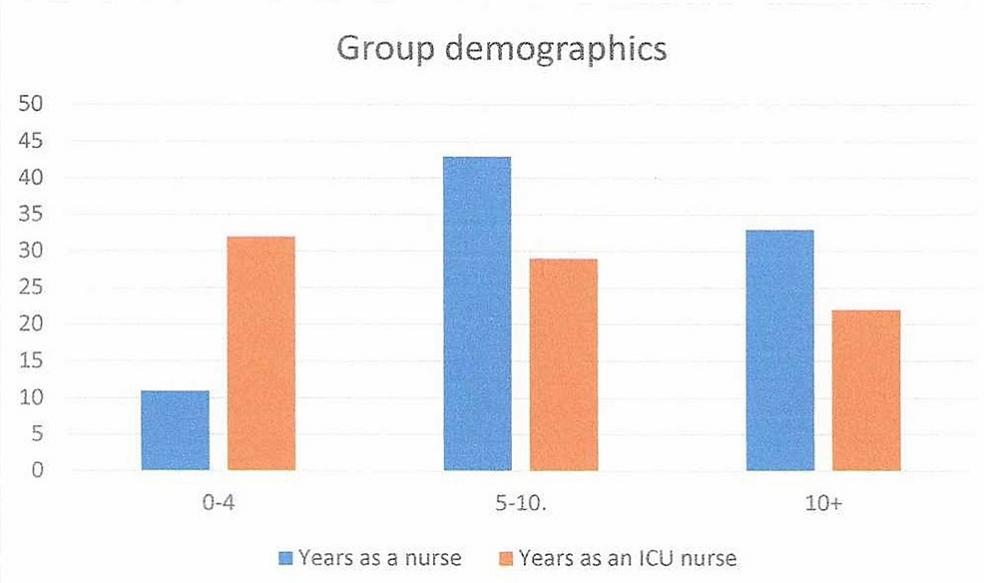
Group demographics N=86.

**Figure 9:**
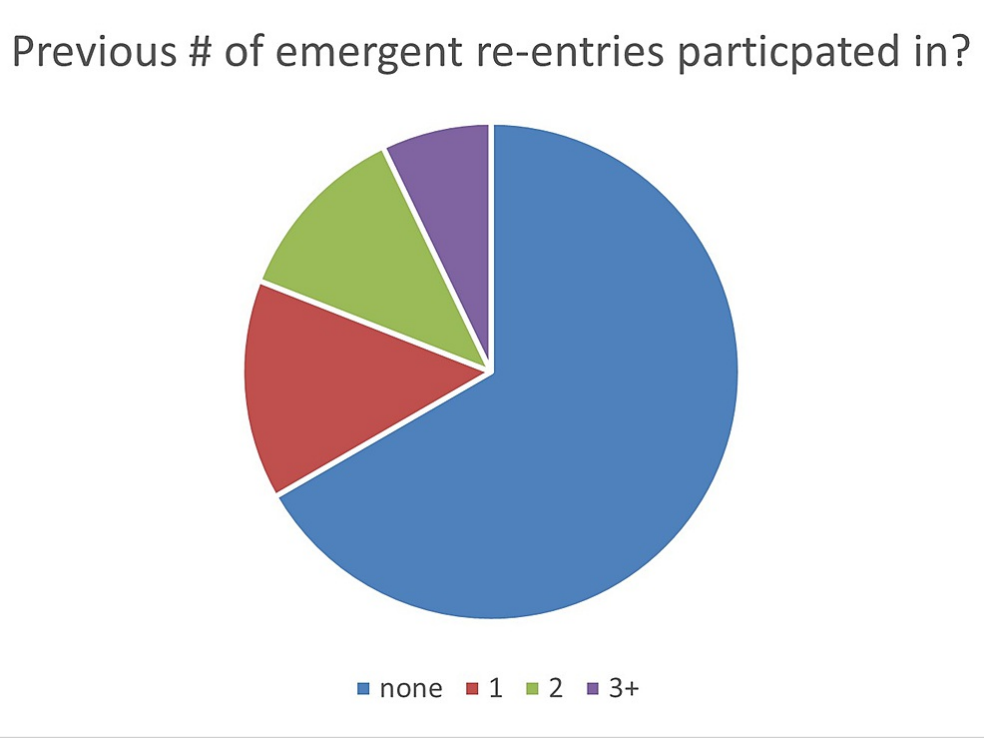
Experience of the group total number of emergent re-entry cases.

**Figure 10:**
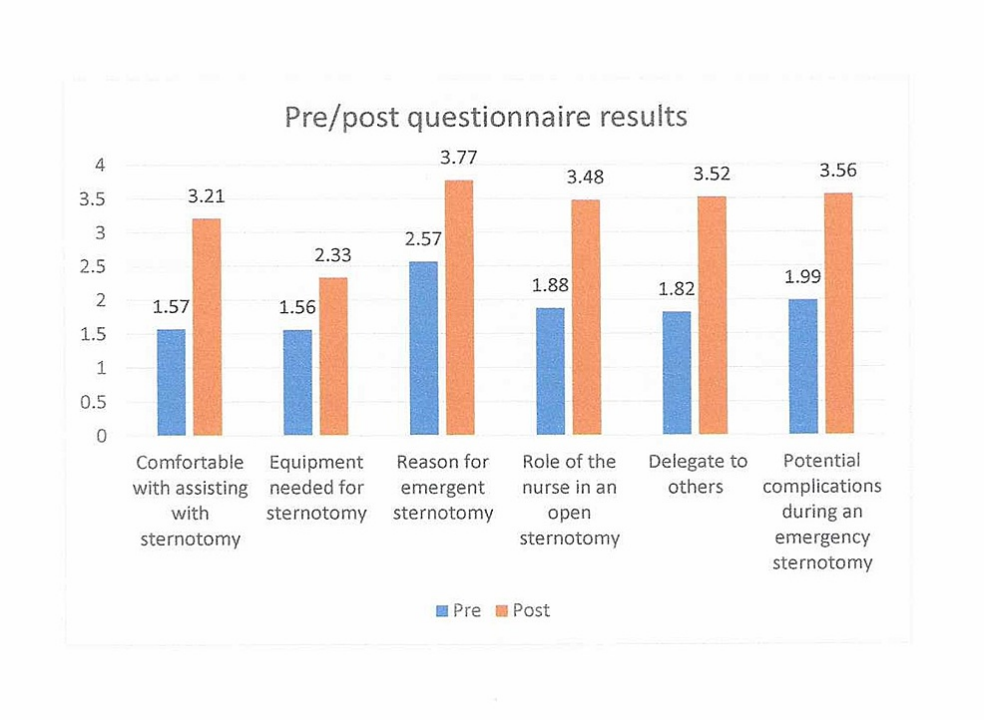
Pre-/post-questionnaire results, N=86; p <0.001 for all six questions.

A pre-questionnaire was developed to measure the demographics of each participant. The measured demographics were years of experience in the participants’ current job title, years of experience working in critical care, and the number of emergent re-sternotomy procedures they participated in during their career. The pre- and post-questionnaire asked the participants about their comfort, knowledge, and proficiency pre- and post-actual hands-on simulation training. Group demographics showed that approximately half of the group (n=43) had been nurses for five to 10 years (Figure 8). The participant’s years of critical care experience were 0-4 years, 5-10 years, and ten plus years, which were 32, 29, and 22. The majority, 56/84 (66%), had never assisted with an emergent re-entry sternotomy procedure (Figure 9). All six questions showed improvement within the pre/post-evaluation. Question 1, “How confident do you feel in assisting with an emergent sternotomy?” improved 1.64, and question 2, “How confident do you feel in knowing what equipment is needed to perform an emergent sternotomy?” improved 1.77. The pre/post-questionnaire results showed improvement ranging from an increase in self-scoring from 1.2 to 1.7, with all questions demonstrating statistical significance with a p <0.001 (Figure 10).

## Discussion

The need to perform/assist with an emergent re-entry sternotomy procedure is a very low-volume, high-risk procedure that we hope not to have happened, but we chose to be prepared in the event it needs to occur. The role of all personnel involved must be understood to increase the chance of survival for the patient. The familiarization of the re-entry cart is another key factor to improving the patient’s chance of survival. All members involved have reported an increase in their level of comfort and proficiency in assisting with an emergent re-sternotomy procedure through this process. Through simulation training, four components, including team participation, repeated process practice and equipment use, and cart familiarization, proved invaluable for increasing overall comfort and proficiency with the skills and knowledge necessary to respond to this type of emergency scenario [5,6,10].

Performing an emergent re-sternotomy for the postoperative cardiac patient is rarely performed in the ICU environment. Furthermore, using a low-fidelity simulation setup did not inhibit the results obtained from this project and positively impacted skill sustainment for low-volume practices. The results obtained from this project practically mirrored the results received using a high-fidelity porcine simulation. The results obtained from this project were statistically comparable to other published results. The previously identified gaps in staff training were addressed by the use of simulation, whether it be low versus high fidelity [1,5,6]. 

Our facility does not have a 24/7 in-house cardiothoracic surgeon on staff. Therefore, if the need for an off-hour emergent re-sternotomy needs to happen, the in-house trauma staff will perform the task of a re-sternotomy. The practice, as mentioned above, represents a high risk to both patients and staff will require our department to modify our resuscitation protocols in the ICU. Also noted are the changes from a single-shock protocol followed by cardiac massage to a three-sequential shock protocol. This change should be discussed in advance as a team responsible for care on the unit. Ideally, these changes and the re-sternotomy procedure simulation training should be given in advance of this practice change. We recommend that all potential individuals caring for cardiac surgical patients practice this protocol regularly and document competencies through simulation training [6,9,11]. The results from this novel approach of using simulation via the SynDaver® mannequin showed that it is feasible to train ICU nurses and providers in emergent re-sternotomies in the ICU environment.

## Conclusions

The results obtained by previous studies utilizing a porcine model were comparable to the results obtained from our simulation approach to assist staff in becoming more comfortable with the various roles of the care team to perform emergent re-entry sternotomy. This project demonstrated that ICU personnel could become more comfortable and confident (1.64, 1.6, and 1.7; p <0.05) to assist with an emergent re-sternotomy. The STS has identified five key factors that should be established in the clinical skills for providing post-cardiac surgery patients, and each of the factors was addressed in our project. The first key factor was utilizing a team approach to rehearse in preparation for post-cardiac surgery arrest situations. The second key factor was obtained from a scenario focused on a post-surgical ventricular fibrillation situation requiring sequential defibrillations before performing external cardiac chest compressions. The third key factor was also obtained from a scenario that required performing immediate treatment of post-surgical asystole or extreme bradycardia via pacing before external cardiac chest compressions were performed. Finally, the fourth key factor was also addressed via a scenario requiring appropriate intervention if pulseless electrical activity. The final key factor was also achieved via inclusion in each scenario written for re-sternotomy training.

The future phase of this project is expected to continue to positively impact the confidence and proficiency levels of the staff that may be involved in emergent re-sternotomy training in the ICU environment over time. The use of simulation was a cost-efficient option for our institution to train staff on the roles of emergent re-entry sternotomy in the post-surgical patient. This project work group, in conjunction with the facilities simulation department, is exploring the various simulation options that are currently on the open market. The team involved with this project intends to also expand the training to include involvement with after-hour simulation scenarios that include surgery department activation and possibly developing an overhead paging alert for simultaneous multi-departmental notification of an emergent re-sternotomy situation.
